# Immunoglobulin G4-related Disease: A New Systemic Disease Emerging in Japan

**DOI:** 10.31662/jmaj.2021-0113

**Published:** 2021-12-15

**Authors:** Terumi Kamisawa

**Affiliations:** 1 Department of Internal Medicine, Tokyo Metropolitan Cancer and Infectious Diseases Center Komagome Hospital, Tokyo, Japan

**Keywords:** IgG4-related disease, IgG4, autoimmune pancreatitis, steroid

## Abstract

Immunoglobulin G4-related disease (IgG4-RD) is a fibro-inflammatory disease characterized by organ enlargement and elevated serum IgG4 levels. In 2003, IgG4-RD was proposed as a distinct form of IgG4-related systemic disease based on a histopathological study involving patients with autoimmune pancreatitis. IgG4-RD occurs mainly in older men and can affect almost any organ simultaneously or metachronously. Pathophysiologically, IgG4-RD occurs when an autoantigen triggers an immune response characterized by Th2 predominance with increased production of cytokines, such as interleukin 4 (IL-4), IL-5, IL-10, IL-13, and tumor growth factor-β (TGF-β), in the affected organ. IL-10 and TGF-β produced by the increased number of regulatory T cells induce a switch from B cells to IgG4-producing plasma cells and fibrosis, respectively. The characteristic histological features consist of dense infiltration of lymphocytes and IgG4-positive plasma cells, storiform fibrosis, and obliterative phlebitis. IgG4-RD is diagnosed based on a combination of clinical, serological, radiological, and histopathological findings. Differentiating IgG4-RD from malignant tumors or similar inflammatory diseases in the affected organs is important. The 2019 America College of Rheumatology/European League against Rheumatism classification criteria for IgG4-RD have high diagnostic sensitivity and specificity. IgG4-RD generally responds well to treatment with steroids, and a swift response is reassuring and provides further diagnostic confirmation. However, relapses are common during tapering or after cessation of steroids. In Japan, low-dose steroid maintenance therapy is usually given to prevent a relapse. B-cell depletion with rituximab is effective in patients resistant to or dependent on steroids. Most patients with IgG4-RD who receive steroid therapy show good short-term clinical, morphological, and functional outcomes. However, long-term outcomes, such as relapse, fibrosis development, and associated malignancies, have not been clearly defined. Therefore, novel treatment strategies, including rituximab, need to be tested in international randomized controlled clinical trials.

## Introduction

Immunoglobulin G4-related disease (IgG4-RD) is an immune-mediated fibro-inflammatory condition characterized by organ enlargement and elevated serum IgG4 levels. It can essentially involve any organ or site simultaneously or metachronously ^(1)^.

A peculiar histological entity of lymphoplasmacytic sclerosing pancreatitis (LPSP) was proposed in 1991 based on a histopathological analysis of a resected specimen of mass-forming pancreatitis that was initially thought to be pancreatic cancer ^(2)^. LPSP is characterized by dense infiltration of lymphocytes and plasma cells with fibrosis in the pancreas. In 1995, the concept of autoimmune pancreatitis (AIP) was proposed based on a case of chronic pancreatitis with hypergammaglobulinemia, which improved with steroid therapy ^(3)^. Hamano et al. reported significant elevation of serum IgG4 levels in patients with AIP in 2001 ^(4)^ and infiltration of IgG4-bearing plasma cells in the pancreas and ureter of three patients with AIP and retroperitoneal fibrosis in 2002 ^(5)^. Kamisawa et al. ^(6)^ detected histopathologically dense fibrosis with abundant infiltration of IgG4-positive plasma cells in the pancreas and other involved organs, such as the peripancreatic tissue, cervical lymph nodes, biliary tract, and salivary gland in seven patients with AIP, and pointed out a close relationship between AIP and multifocal fibrosclerosis ^(7)^, an uncommon fibroproliferative systemic disorder with multiple manifestations, including retroperitoneal fibrosis, sclerosing cholangitis, fibrotic orbital pseudotumor, and salivary gland fibrosis. Furthermore, they found abundant IgG4-positive plasma cell infiltration in various organs and sites, such as the liver, stomach, and colon, in patients with AIP ^(8)^. On the basis of these findings, in 2003, the authors proposed “IgG4-related systemic disease” as a new clinicopathological systemic entity ^(8), (9)^.

In 1892, Mikulicz et al. described a patient with symmetrical enlargement of the lachrymal, parotid, and submandibular glands with massive infiltration of mononuclear cells ^(10)^, and the condition was called Mikulicz’s disease. However, Mikulicz’s disease has been classified in the West as an atypical type of Sjögren’s syndrome. Yamamoto et al. reported elevated serum IgG4 levels in patients with Mikulicz’s disease in 2004 ^(11)^. In 2005 ^(12)^, they reported an infiltration of numerous IgG4-positive plasma cells in the lacrimal and salivary glands of patients with Mikulicz’s disease and pointed out some clinical differences between Mikulicz’s disease and Sjögren’s syndrome, including the sex ratio and the degree of keratoconjunctivitis sicca and steroid responsiveness. On the basis of these findings, they proposed that Mikulicz’s disease is systemic and renamed it systemic IgG4 plasmacytic syndrome in 2006 ^(13)^. In 2010, these various entities were unified under the single term, IgG4-RD, by Japanese research teams ^(14)^.

No simple diagnostic test currently exists for IgG4-RD, which comprises many conditions previously thought to be isolated, single-organ diseases with no known underlying systemic disease. Further, IgG4-RD often presents with findings that mimic malignancy both clinically and radiologically. Therefore, diagnosing IgG4-RD remains a major clinical challenge. To provide the appropriate therapy, it is critically important to differentiate IgG4-RD from malignant tumors and similar inflammatory diseases in the affected organ in a timely and accurate manner ^(1)^.

## Epidemiology

As IgG4-RD is relatively rare and not well known, its epidemiology is difficult to ascertain. A nationwide epidemiological survey of AIP in Japan in 2016 ^(15)^ estimated that 13,436 patients had AIP and that the overall prevalence of cases was 10.1 per 100,000 population. The estimated number of newly diagnosed cases was 3,984, with an annual incidence of 3.1 cases per 100,000 population. Both numbers had more than doubled over the figures in the 2011 survey. This apparent increase may be attributable to more widespread awareness of the disease concept and refinements in the diagnostic criteria. The male-to-female sex ratio was 2.94, and the mean age at diagnosis was 64.8 years. IgG4-RD occurs mainly in older men.

## Pathophysiology

Multiple pathogenic factors have been proposed as playing a key role in IgG4-RD development, but the pathogenic mechanism of the disease remains unclear. Initially, autoantigens trigger an immune response characterized by Th2 predominance and increased production of cytokines, such as interleukin 4 (IL-4), IL-5, IL-10, and IL-13, as well as tumor growth factor-β (TGF-β) in the affected organ. IL-10 and TGF-β produced by the increased number of regulatory T cells induce the switch from B cells to IgG4-producing plasma cells and fibrosis, respectively. IgG4 itself does not seem to be a driver of the pathogenesis ^(16), (17)^.

Abnormal innate immunity has also been demonstrated in some patients with IgG4-related disease. Watanabe et al. reported that activation of nucleotide-binding oligomerization domain-2 and Toll-like receptor ligands on monocytes or basophils from patients with AIP enhanced the IgG4 response via B-cell activating factor and IL-13 ^(18), (19)^.

Shiokawa et al. reported that laminin-511 is a target antigen in AIP. An autoantibody against laminin-511 was identified in 26 (51%) of 51 patients with AIP, and immunization of mice with human laminin-511 induced antibodies and pancreatic injury fulfilling the pathological criteria for human AIP ^(20)^.

## Pathology

**Figure 1.**
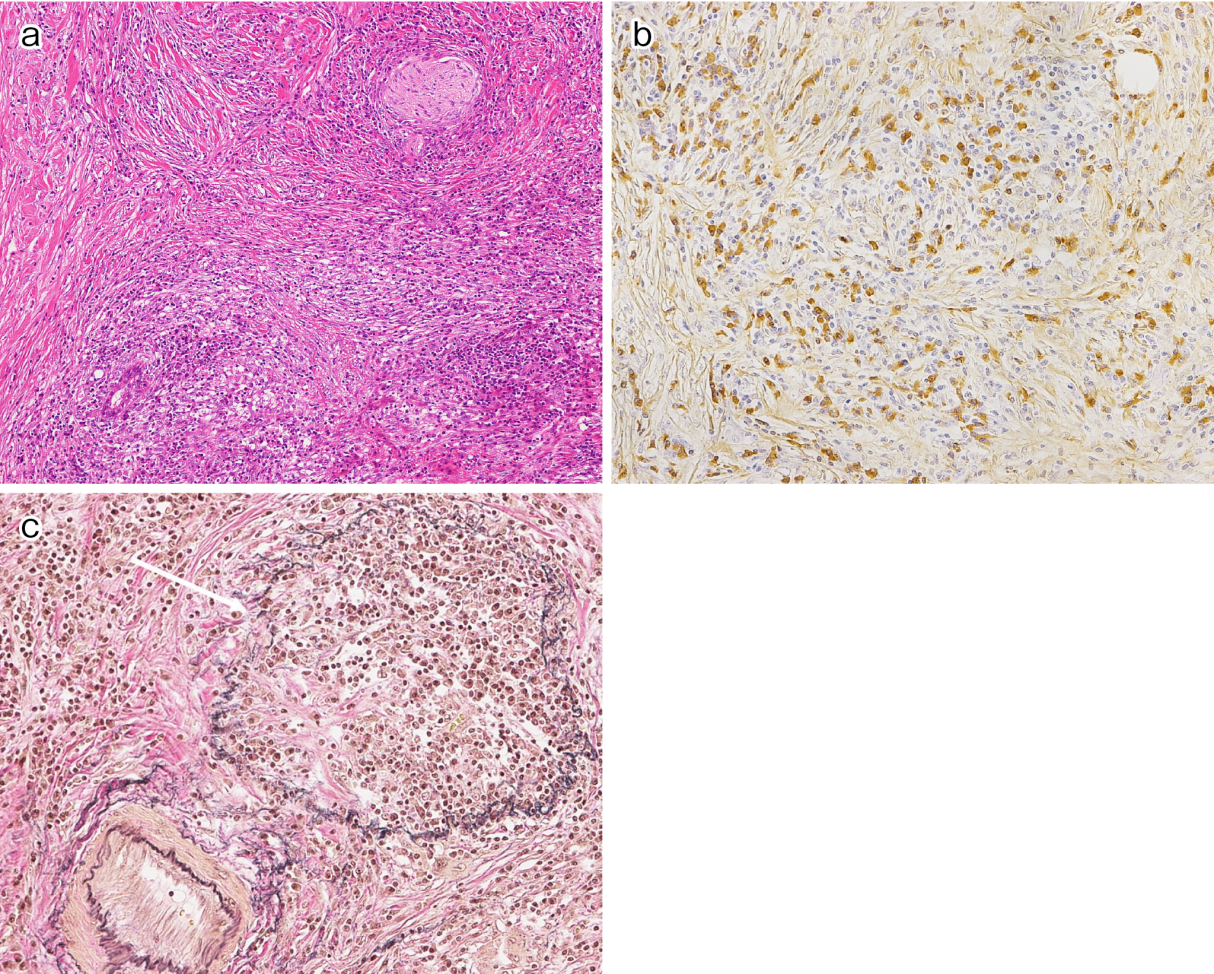
Histopathological features of IgG4-related disease. (a) Dense infiltration of lymphocytes and plasma cells with storiform fibrosis (H&E staining). (b) Abundant infiltration of IgG4-positive plasma cells (IgG4 immunostaining). (c) Obliterative phlebitis (arrow, Elastica van Gienson staining).

Together with storiform fibrosis and obliterative phlebitis, dense infiltration of lymphocytes and plasma cells that are immunohistochemically positive for anti-IgG4 antibody are the characteristic histopathological features of IgG4-RD (Figure 1a, b and c). Storiform fibrosis shows an irregular, whorled pattern of radially arranged collagen fibers. Obliterative phlebitis is defined as either partial or complete obliteration of medium-sized veins that can sometimes be identified only with elastic staining. However, both storiform fibrosis and obliterative phlebitis are rare in the lacrimal glands and lymph nodes.

At present, the characteristic histology and immunohistochemistry of IgG4-RD represent the gold standard of diagnosis. However, biopsies often provide an insufficient amount of tissue for diagnostic confirmation. In addition, significant infiltration of IgG4-positive plasma cells in biopsied specimens is not specific to IgG4-RD, and this has been reported in other conditions commonly known to mimic IgG4-RD, including malignancies. Semiquantitative analysis of IgG4 immunostaining may help in distinguishing IgG4-RD from similar conditions. A frequently used cutoff value for IgG4-positive plasma cell infiltration is >10 cells per high power field, but this varies by tissue. Evaluating the ratio of IgG4-positive/total IgG-positive cells, typically with a minimum ratio of 40%, is also reportedly useful ^(1), (21)^.

Although the findings of storiform fibrosis and obliterative phlebitis can enhance diagnostic specificity, correlation with clinicopathological findings is essential for a definitive diagnosis ^(1), (21)^. Samples from previously biopsied or resected specimens may be useful if they are reviewed along with the IgG4-immunostaining of paraffin-embedded specimens.

The pathological exclusion criteria of the American College of Rheumatology/European League Against Rheumatism (ACR/EULAR) classification criteria for IgG4-RD (Table 1) ^(22)^ are as follows: cellular infiltrates suspicious for malignancy that have not been investigated sufficiently, markers consistent with inflammatory myofibroblastic tumor, prominent neutrophilic infiltration, necrotizing vasculitis, prominent necrosis, primary granulomatous inflammation, and pathologic features of a macrophage/histiocytic disorder.

**Table 1.** The 2019 American College of Rheumatology/European League Against Rheumatism Classification Criteria for IgG4-Related Disease ^(18)^.

Step	Categorical assessment or numeric weight
**Step 1. Entry criteria**	**Yes** or No
Characteristic clinical or radiologic involvement of a typical organ OR pathologic evidence of an inflammatory process accompanied by a lymphoplasmacytic infiltrate of uncertain etiology	
**Step 2. Exclusion criteria: domains and items**	Yes or **No**
*Clinical*	
Fever	
No objective response to glucocorticoid	
Serologic	
Leukopenia and thrombocytopenia with no explanation	
Peripheral eosinophilia	
Positive antineutrophil cytoplasmatic antibody	
Positive SSA/Ro or SSB/La antibody	
Positive double-stranded DNA, RNP, or Sm antibody	
Other disease-specific autoantibody	
Cryoglobulinemia	
*Radiologic*	
Known radiologic findings suspicious for malignancy or infection that have not been sufficiently investigated	
Rapid radiologic progression	
Long bone abnormalities consistent with Erdheim-Chester disease	
Splenomegaly	
*Pathologic*	
Cellular infiltrates suggesting malignancy that have not been sufficiently investigated	
Markers consistent with inflammatory myofibroblastic tumor	
Prominent neutrophilic infiltration	
Necrotizing vasculitis	
Prominent necrosis	
Primarily granulomatous infiltration	
Pathologic features of macrophage/histiocytic disorder	
*Known diagnosis of the following:*	
Multicentric Castleman’s disease	
Crohn’s disease or ulcerative colitis (if only pancreatobiliary disease is present)	
Hashimoto thyroiditis (if only the thyroid is affected)	
**Step 3. Inclusion criteria: dominant and items**	
*Histopathology*	
Dense lymphocytic infiltrate	+4
Dense lymphocytic infiltrate and obliterative phlebitis	+6
Dense lymphocytic infiltrate and storiform fibrosis with or without obliterative phlebitis	+13
Immunostaining	
The IgG4+:IgG+ ratio and number of IgG4+ cells/hpf	+0-16
Serum IgG4 concentration	
Normal but < 2x upper limit of normal	+4
2-5x upper limit of normal	+6
>5x upper limit of normal	+11
Bilateral lacrimal, parotid, sublingual, and submandibular glands	
One set of glands involved	+6
Two or more sets of glands involved	+14
Chest	
Peribronchovascular and septal thickening	+4
Paravertebral band-like soft tissue in the thorax	+10
Pancreas and biliary tree	
Diffuse pancreas enlargement (loss of lobulations)	+8
Diffuse pancreas enlargement and capsule-like rim with decreased enhancement	+11
	+19
Pancreas (either of above) and biliary tree involvement
Kidney	+6
Hypocomplementemia	+8
Renal pelvis thickening/soft tissue	+10
Bilateral renal cortex low-density areas	
Retroperitoneum	+4
Diffuse thickening of the abdominal aortic wall	+8
Circumferential or anterolateral soft tissue around the infrarenal aorta iliac arteries	
**Step 4: Total inclusion points**
**A case meets the classification criteria for IgG4-RD if the entry criteria are met, no exclusion criteria are present, and the total points is ≥20.**

## Clinical Symptoms and Affected Organs

IgG4-RD is a systematic disease affecting various organs with organomegaly or hypertrophy. The clinical symptoms of IgG4-RD depend on the individual pattern of organ involvement and the severity of disease activity. Patients with IgG4-RD sometimes have a history of allergic disease or atopic features ^(23)^.

Most cases follow a subacute or chronic course, whereas others show spontaneous improvement ^(24)^. Severe constitutional symptoms are rare, but organomegaly or hypertrophy can occasionally lead to serious complications, such as obstruction or compression, including obstructive jaundice, visual disturbance, and hydronephrosis in AIP or IgG4-related sclerosing cholangitis (IgG4-SC), IgG4-related dacryoadenitis, and IgG4-related retroperitoneal fibrosis, respectively. In addition, there are previous reports of persistent inflammation in the affected organs resulting in fibrosis and permanent organ dysfunction or failure ^(25)^. The organs most frequently involved are the pancreas and biliary tract, followed by the salivary glands, kidneys, and lachrymal glands ^(26)^.

## Diagnosis

### Diagnostic criteria

Diagnosis relies on a combination of various clinical, laboratory, radiological, and histopathological findings. Specific diagnostic criteria have been developed for several organs, and comprehensive diagnostic criteria for IgG4-RD ^(27)^ independent of predominant organ involvement have also been proposed. The 2019 ACR/EULAR classification criteria for IgG4-RD (Table 1) consist of entry, exclusion, and inclusion criteria and have high sensitivity and specificity ^(22)^.

### Laboratory data

Serum IgG4 levels are frequently elevated in patients with IgG4-RD. In a previous study, elevated serum IgG4 levels (≥ 135 mg/dL) were reported in 84% (1586/1883) of patients with IgG4-RD (mean: 769 mg/dL) ^(28)^. In serological testing, polyclonal hypergammaglobulinemia, elevated immunoglobulin E levels, the presence of antinuclear antigen, and the presence of rheumatoid factor were found in 61%, 58%, 30%, and 20% of patients with IgG4-RD, respectively ^(28)^. Hypocomplementemia was found in 53% of patients with IgG4-related kidney disease ^(29)^.

ACR/EULAR serological exclusion criteria have been developed for leukopenia and thrombocytopenia with no explanation, peripheral eosinophilia, and positive disease-specific autoantibodies ^(22)^.

### Imaging

Computed tomography (CT), magnetic resonance imaging (MRI), and ^18^F-fluorodeoxyglucose positron emission tomography (PET) are useful in diagnosing IgG4-RD. Diffuse or focal swelling of organs or soft tissue masses appears with soft tissue attenuation, well-defined margins, and homogeneous, late-stage enhancement on enhanced CT images of IgG4-RD ^(30)^.

### Steroid trial

IgG4-RD responds well to steroids, so a swift response to steroids can help in confirming the presence of IgG4-RD in patients with collateral evidence. A steroid trial can be performed if enough biopsy specimens cannot be obtained and differentiating IgG4-RD from malignancy is difficult. However, a careful, diagnostic steroid trial should only be conducted after a negative work-up for malignancy that includes a histopathological approach or in cases where imaging modalities can be used to evaluate the effects of steroid therapy because symptomatic and hematological improvement can occur nonspecifically in response to steroids, even in cases of malignancy ^(31), (32)^.

## Main Organ Manifestations

### Autoimmune pancreatitis (AIP)

**Figure 2.**
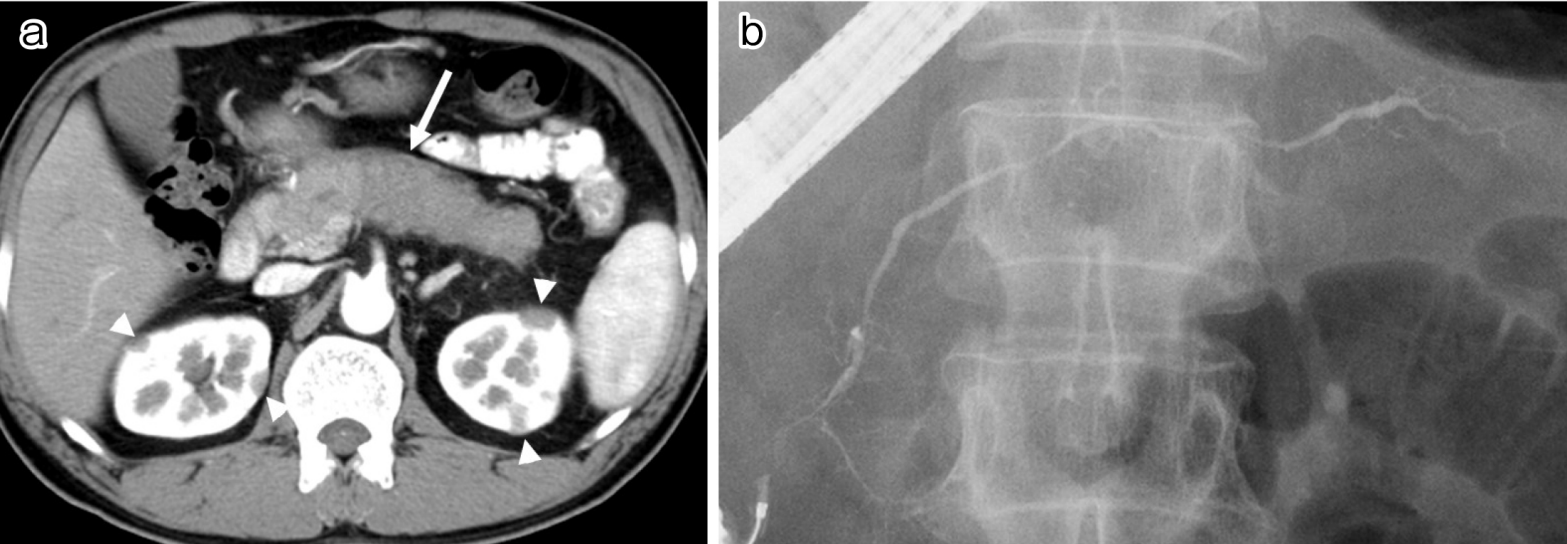
Images of autoimmune pancreatitis. (a) Diffuse enlargement of the pancreas (arrow) and multiple low-density areas in the kidney (arrowheads) on enhanced CT. (b) Diffuse, irregular narrowing of the main pancreatic duct on endoscopic retrograde pancreatography.

In AIP, the pancreatic duct epithelium is well preserved despite the surrounding inflammation ^(30)^. The common clinical presentation of this disease involves painless obstructive jaundice (49%) and abdominal pain (26%), with about one-third of patients being asymptomatic ^(15)^. The International Consensus Diagnostic Criteria ^(32)^ recommend a combination of serum IgG4 level, characteristic imaging, other organ involvement, histology, and steroid responsiveness. Characteristic imaging findings of AIP show diffuse enlargement of the pancreas with delayed enhancement on enhanced CT (Figure 2a) accompanied by diffuse irregular narrowing of the main pancreatic duct on endoscopic retrograde pancreatography (Figure 2b). A capsule-like, low-density rim surrounding the pancreas is rather specific to AIP. Differentiation from pancreatic cancer can be difficult in atypical cases with a localized, short stricture of the main pancreatic duct or segmental/focal enlargement of the pancreas. At present, a histological approach involving endoscopic ultrasonography (EUS)-guided fine needle aspiration is employed in about half of the patients with AIP in Japan. However, specimens are sometimes too small for histological diagnosis, so novel types of EUS-guided needles are needed to obtain pathological samples of a sufficient size ^(1), (30)^.

### IgG4-related sclerosing cholangitis (IgG4-SC)

**Figure 3.**
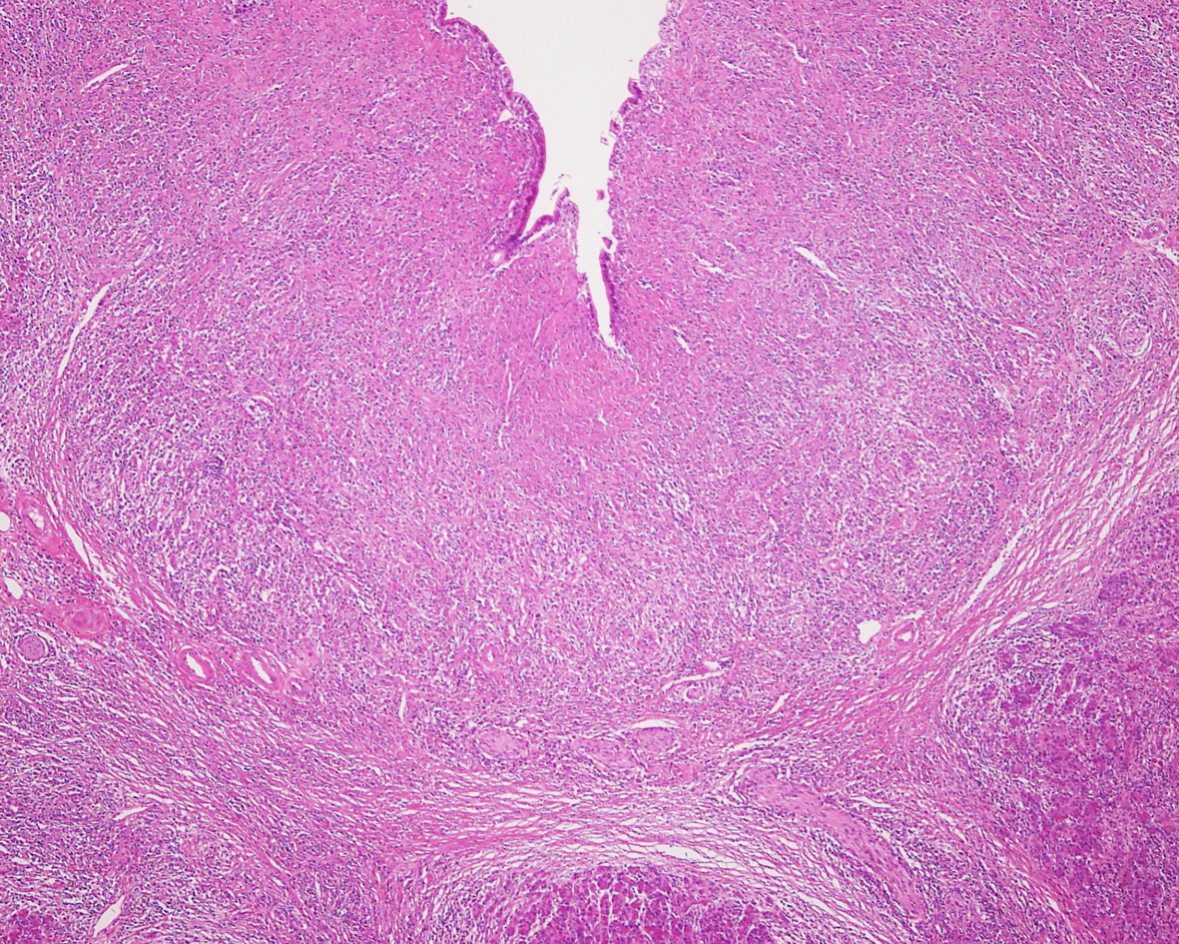
Histological finding of IgG4-related sclerosing cholangitis showing transmural wall thickening with fibrosis and lymphoplasmacytic infiltration (H&E staining).

AIP is the most commonly reported comorbidity (84%) in IgG4-SC ^(33)^, whereas IgG4-SC is associated with 49% of AIP cases ^(15)^. In patients with IgG4-SC, jaundice is the most common presenting symptom, followed by pruritus and abdominal pain. However, 37% of cases are diagnosed in the absence of any symptoms ^(33)^. Histologically, transmural wall thickening with fibrosis and lymphoplasmacytic infiltration is observed (Figure 3) ^(31)^.

**Figure 4.**
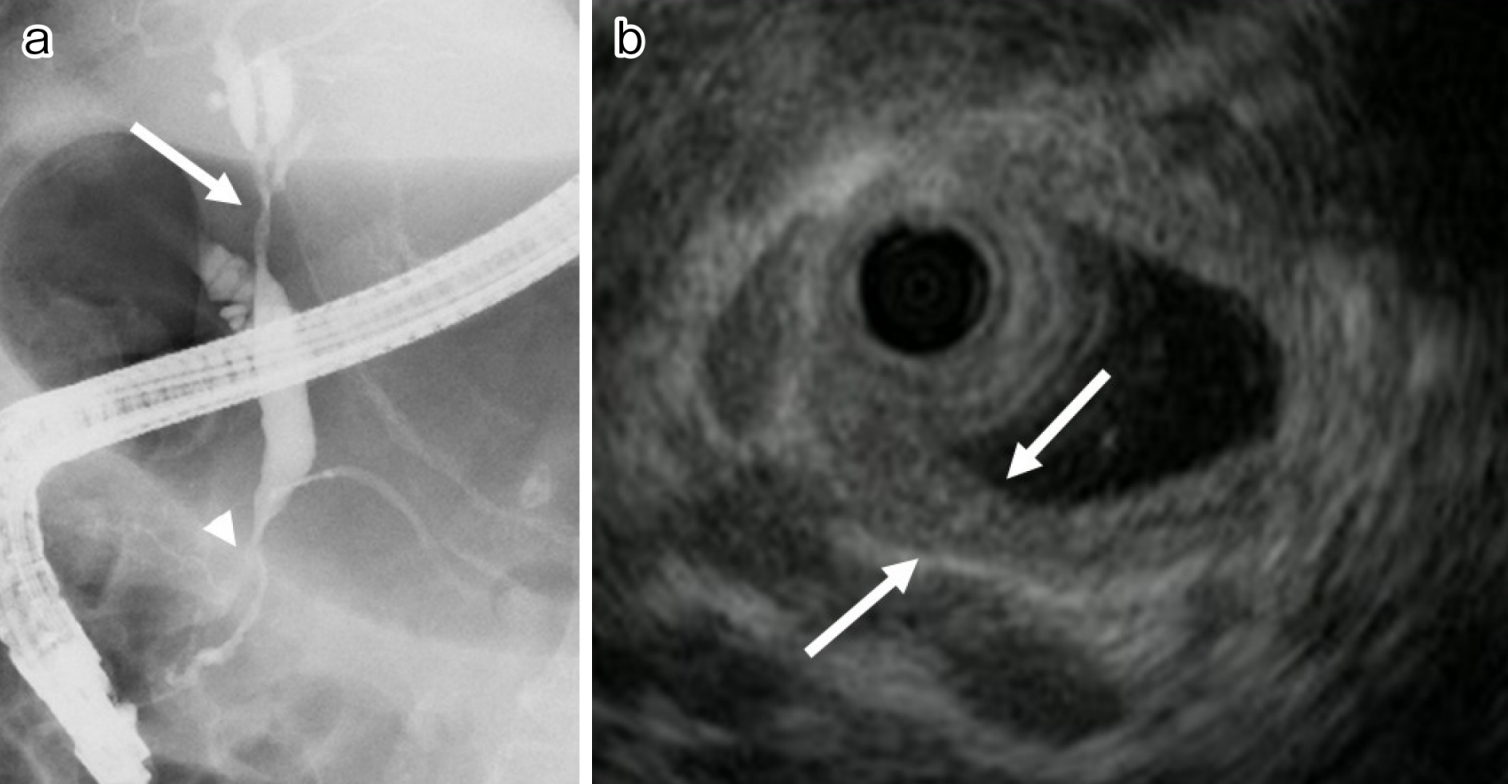
Images of IgG4-related sclerosing cholangitis. (a) Stenosis of the hilar (arrow) and lower (arrowhead) bile duct on endoscopic retrograde cholangiopancreatography. (b) Intraductal ultrasonography showing extensively symmetrical wall thickening with a smooth inner and outer margin that is not stenotic on cholangiography (arrows).

In IgG4-SC, stenosis of the lower bile duct occurs most frequently. When the hilar and/or intrahepatic bile ducts are involved, differentiating IgG4-SC from primary sclerosing cholangitis (PSC) and cholangiocarcinoma is crucial (Figure 4a). In IgG4-SC, the bile duct epithelium is preserved unlike in PSC or cholangiocarcinoma. Cholangiographic findings are helpful in differentiating IgG4-SC from PSC, but cholangiography alone cannot distinguish IgG4-SC from cholangiocarcinoma. Intraductal ultrasonography (IDUS) can be more helpful in differentiating IgG4-SC from cholangiocarcinoma. IDUS findings highly suggestive of IgG4-SC include circular, symmetrical wall thickening, smooth inner or outer margin, homogeneous internal echo, preservation of the three layers of the stenotic area, and extensive thickening of the bile duct wall that appears normal on cholangiography (Figure 4b). IDUS findings suggestive of cholangiocarcinoma include eccentric wall thickening with an irregular luminal surface, disruption of the layered structure of the bile duct wall, and a hypoechoic mass with irregular margins ^(31), (34)^.

### IgG4-related hepatopathy and IgG4-related autoimmune hepatitis

Two types of hepatic parenchymal involvement have been reported in IgG4-RD. IgG4-related hepatopathy presents as heterogenous lesions in the liver parenchyma of patients with AIP or IgG4-SC. Histological findings include portal inflammation, large bile duct damage, portal sclerosis, lobular hepatitis, and cholestasis, which can be explained as the secondary cause of sclerosing cholangitis or obstruction of the extrahepatic biliary tract ^(35), (36)^.

IgG4-related autoimmune hepatitis is clinicopathologically similar to classical autoimmune hepatitis with high-grade necro-inflammatory activity, except for elevated serum IgG4 levels and increased IgG4-positive plasma cell infiltration in the liver ^(36), (37)^.

### IgG4-related gastrointestinal disease

Two types of IgG4-related gastrointestinal disease have been identified. The first one involves a gastrointestinal lesion accompanied by marked thickening of the esophageal and gastric walls consisting of dense fibrosis with abundant IgG4-positive plasma cell infiltration, typically with submucosal spread. The second one is IgG4-related pseudotumor with a polypoid or mass-like form occurring in the stomach, colon, or major duodenal papilla ^(38)^.

Notohara et al. reported that IgG4-related gastrointestinal disease is found in the context of IgG4-RD. Two unique histological patterns are striated inflammatory lesions in the muscularis propria giving rise to thickening and bottom-heavy plasmacytosis in the mucosa ^(39)^.

### IgG4-related dacryoadenitis and sialadenitis

Both Mikulicz’s disease, which consists of bilateral symmetrical swelling of the lacrimal and salivary glands, and Küttner’s tumor, a type of chronic sclerosing sialadenitis presenting with firm, asymmetrical swelling of the submandibular gland, are currently recognized as forms of IgG4-RD ^(1), (14)^. Newly proposed diagnostic criteria for IgG4-related dacryoadenitis and sialadenitis ^(40)^ include symmetrical swelling of the lachrymal, parotid, or submandibular glands for at least three months along with sonographic features of the submandibular glands, such as “hypoechoic areas of the nodal pattern with high vascularity” and/or “hypoechoic areas of reticular patterns surrounding normal parenchyma.” Differential diagnosis is necessary to distinguish IgG4-related dacryoadenitis and sialadenitis from Sjögren’s syndrome, sarcoidosis, Castleman disease, Wegener’s granulomatosis, lymphoma, and cancer. Fewer sicca symptoms are seen, and anti-SSA and SS-B antibodies are rarely positive. In IgG4-related sialadenitis, the submandibular glands are more commonly affected than the parotid glands ^(1)^.

**Figure 5.**
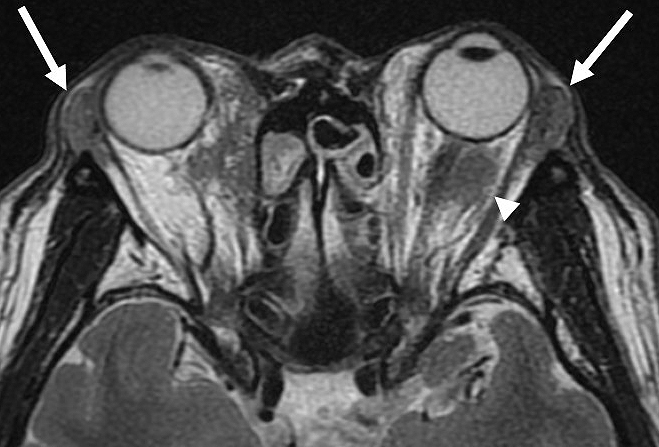
MRI of IgG4-related dacryoadenitis showing bilateral swelling of lacrimal glands (arrows) and retrobulbar inflammation (arrowhead).

In IgG4-related dacryoadenitis, other tissues besides the lacrimal glands, such as extraocular muscles, orbital fat tissue, the eyelids, the trigeminal nerve, and the nasolacrimal duct, may be involved (Figure 5). Clinical symptoms include eyelid swelling, diplopia, ptosis, visual field disturbance, eye pain, decreased visual acuity, eye movement disturbance, dry eye, corneal ulcer, and epiphora ^(41), (42)^.

### IgG4-related rhinosinusitis

IgG4-RD also involves nasal manifestations with chronic rhinosinusitis. Moteki et al. reported that IgG4-related rhinosinusitis occurred in 32% of 31 patients with IgG4-RD ^(43)^. Hanaoka et al. ^(44)^ reported that all 10 patients in their study with IgG4-related rhinosinusitis also had multiple forms of IgG4-RD. The clinical symptoms of IgG4-related chronic rhinosinusitis include nasal obstruction, nasal discharge, postnasal discharge, hyposmia, and dull headache. Radiological findings include obstruction of the way of elimination, thickening of the sinus mucous membrane, and fluid in the sinus. A deviated nasal septum, polyps, and edema of the mucous membrane are frequently seen in nasal endoscopy.

### IgG4-related thyroid disease

Patients with AIP are highly positive for antithyroglobulin antibodies and susceptible to hypothyroidism ^(45)^. Shimizuguchi et al. ^(46)^ detected hypothyroidism in 8 (10%) of 77 patients with AIP and subclinical and central hypothyroidism in six and two patients, respectively. Thyroid diseases associated with IgG4 include autoimmune thyroid disease and Riedel’s thyroiditis. According to the 2021 diagnostic criteria for IgG4-related thyroid disease ^(47)^, IgG4-related thyroiditis is diagnosed definitively if all of the following criteria are met: enlargement of the thyroid, hypoechoic lesions in the thyroid on ultrasound, elevated serum IgG4 levels, and histopathological findings of the thyroid lesion. IgG4-related thyroiditis is frequently confused with a malignancy because of the intense sclerosis of the thyroid, resulting in a hard texture on palpation compounded frequently by associated compressive symptoms.

### IgG4-related hypophysitis

The main symptoms of IgG4-related hypophysitis result from a mass lesion in the pituitary gland, hypopituitarism, or from central diabetes insipidus. Also frequently found are decreased levels of one or more hormones from the anterior pituitary gland or the affected organs, decreased response of anterior pituitary hormones on a stimulation test, and diffuse enlargement of the pituitary gland and/or stalk on imaging studies ^(48)^.

### IgG4-related periaortitis/periarteritis and retroperitoneal fibrosis

**Figure 6.**
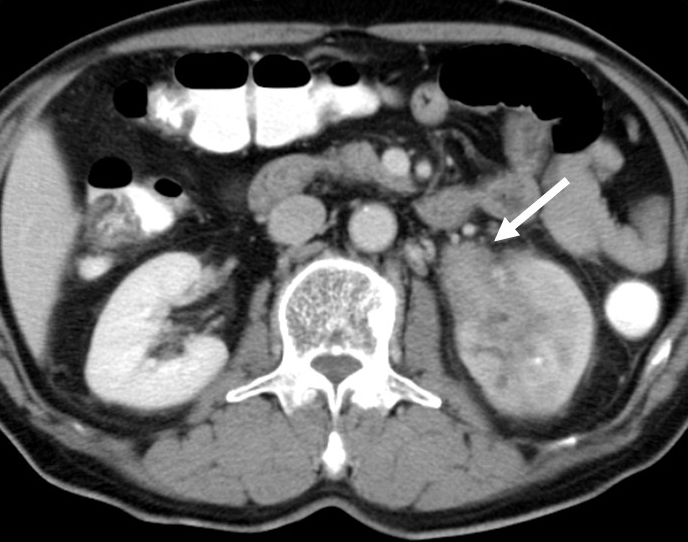
Enhanced CT of IgG4-related retroperitoneal fibrosis showing a soft tissue mass in the renal pelvis (arrow).

IgG4-RD is reportedly the cause of as many as two-thirds of all cases of idiopathic retroperitoneal fibrosis, also known as Ormond’s disease ^(1), (49)^. Low-density hypertrophic thickening or soft tissue masses around the aorta and its major branches, sometimes accompanied by luminal dilatation or aneurysm formation, and soft tissue masses in the renal pelvic wall that are sometimes associated with hydronephrosis are characteristic abdominal radiological findings of IgG4-related periaortitis/periarteritis and retroperitoneal fibrosis (Figure 6). It is important to exclude atherosclerosis, arterial dissection, infectious disease, other forms of vasculitis, and malignant diseases such as malignant lymphoma and cancers. The clinical presentation can be subtle and nonspecific, leading to delays in diagnosis. Common presentations include poorly localized pain in the back, flanks, lower abdomen, thighs, leg edema, and hydronephrosis due to ureteral involvement ^(50), (51)^.

Severe complications include aneurysm formation, vessel rupture, and renal dysfunction resulting from hydronephrosis. Although the first two complications are known to occur in the absence of steroid therapy ^(52)^, rupturing of affected vessels after steroid therapy has also been reported ^(53)^. The risks and benefits of steroid therapy for periarterial lesions remain controversial ^(51)^.

As hydronephrosis caused by IgG4-related retroperitoneal fibrosis is known to lead to irreversible renal impairment, starting steroid therapy early in combination with urological surgical interventions, such as stent placement or nephrostomy, is important, depending on the presence of a urinary tract infection or renal impairment ^(25), (51)^.

### IgG4-related lung disease

IgG4-related respiratory disease is associated with a limited number of subjective symptoms and is usually detected in patients with extra-thoracic IgG4-RD lesions during work-up. In some patients, IgG4-related respiratory disease can manifest nonspecific clinical symptoms, which can delay the diagnosis. On chest CT, the most common abdominal shadow is hilar and mediastinal lymphadenopathy, followed by thickening of the bronchial wall, bronchovascular bundles, and interlobular septal wall, nodular and infiltrative shadows, and pleural thickening/effusion ^(54), (55)^.

### IgG4-related kidney disease

The most representative lesion in IgG4-related kidney disease is plasma cell-rich tubulointerstitial nephritis (TIN); membranous glomerulonephritis is also a representative glomerular lesion. Decreased renal function, mild proteinuria, and mild hematuria are typical clinical features of IgG4-related kidney disease, but most kidney lesions are incidentally detected during the examination for other organ involvement using contrast-enhanced CT in patients already known to have IgG4-RD. Abnormal renal radiological findings include multiple, low-density lesions on enhanced CT (Figure 2b); diffuse kidney enlargement; a round, hypovascular, solitary mass in the kidney; and hypertrophic lesions of the renal pelvic wall without an irregular renal pelvic surface ^(56), (57), (58)^. As treatment delay is associated with partial scarring of the kidneys on imaging studies, steroid therapy should be initiated as soon as possible after IgG4-TIN is diagnosed ^(25), (56)^.

### IgG4-related lymphadenopathy

Enlarged lymph nodes are usually asymptomatic and therefore sometimes detected incidentally on imaging examinations. Lymph node lesions in IL-6 syndromes, such as plasma cell-type Castleman disease and rheumatoid arthritis, mimic IgG4-related lymphadenopathy ^(59), (60)^. Satou et al. suggested that IgG4-related lymphadenopathy consists of five histological subtypes and proposed exclusion criteria to distinguish them as follows: continued elevation of serum CRP levels, elevated serum IgA and IgM levels, pathological findings of a sheet-like proliferation pattern of mature plasma cells, high degree of hemosiderin deposition, and neutrophilic infiltration ^(61)^.

### Other organs

IgG4-related hypertrophic pachymeningitis is restricted to the dura and causes headaches by its traction, as well as irritation of the trigeminal nerve and higher central roots ^(62)^.

IgG4-related cholecystitis is seen in about half of patients with IgG4-SC as a diffuse thickening of the gallbladder wall with a smooth inner surface ^(63)^.

IgG4-related skin lesions present as erythematous, itchy plaques, or subcutaneous nodules of the head and neck, particularly in the periauricular, cheek, and mandibular areas, which are frequently accompanied by other extracutaneous forms of IgG4-RD ^(64)^.

IgG4-related prostatitis is occasionally accompanied by IgG4-RD in other organs and is detected incidentally on PET. Some patients experience a resolution of urinary symptoms after steroid treatment ^(65)^.

IgG4-related mastitis is an IgG4-related inflammatory pseudotumor of the breast and should be differentiated radiologically from breast cancer and histologically from mucosa-associated lymphoid tissue lymphoma ^(66)^.

IgG4-related mesenteritis shows a hypoattenuating mass with or without encasement of mesenteric vessels in the mesentery, and its preoperative diagnosis is quite difficult ^(67)^.

In IgG4-related mediastinitis, compression of vital, mediastinal structures can result from proliferation of invasive fibrous tissue within the mediastinum ^(68)^.

## Treatment

### Steroid therapy

**Figure 7.**
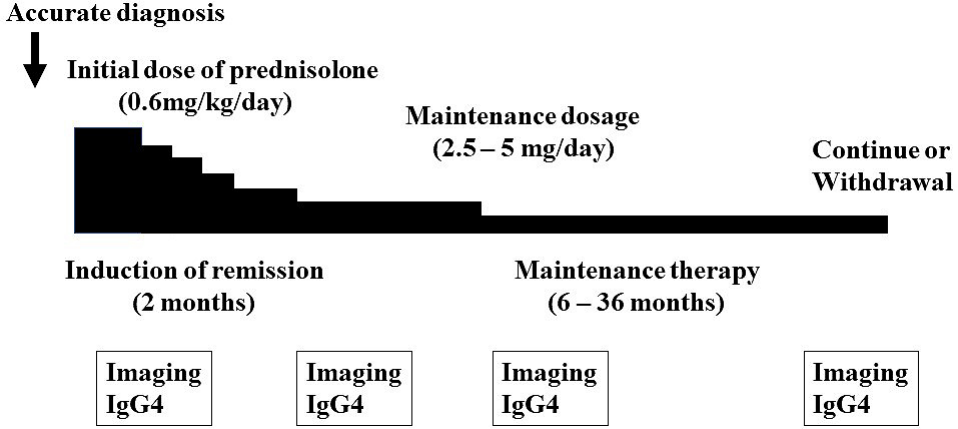
Regimen of oral steroid therapy for IgG4-related disease.

It is essential that an accurate diagnosis of IgG4-RD is confirmed before starting steroid treatment. Steroids are the first-line treatment for IgG4-RD. The Japanese guidelines ^(24)^ recommend prednisolone 0.6 mg/kg/day (usually 30-40 mg per day) for three months to induce remission. Clinical improvement after the start of steroid therapy is typically rapid, so follow-up serological and imaging assessments should be conducted about two weeks later. As patients with IgG4-RD frequently experience a relapse during steroid tapering or cessation, maintenance therapy with low-dose prednisolone (2.5-5 mg per day) for 6-36 months is usually given in Japan to maintain remission and prevent relapses (Figure 7). A retrospective, long-term study of 510 patients with AIP ^(69)^ and a randomized controlled trial ^(70)^ showed that low-dose maintenance steroid treatment was effective in reducing the relapse rate in patients with AIP. A poor response to steroid therapy should increase the possibility of another diagnosis, particularly cancer. The response to steroid can vary depending on the affected organ and degree of fibrosis. If left uncontrolled, several types of IgG4-RD can lead to irreversible damage to certain organs, such as aortitis, retroperitoneal fibrosis, sclerosing cholangitis, TIN, pachymeningitis, and pericarditis ^(25)^. Thus, urgent treatment is recommended in such cases.

### Immunosuppressive drugs

Steroid readministration or dose-increase has been shown to be effective in cases of relapsed IgG4-RD. When the steroid dose cannot be tapered because of a persistently active disease, the addition of immunosuppressants, such as azathioprine, mycophenolate mofetil, or 6-mercaptopurine, is appropriate ^(24)^. However, a retrospective study comparing immunosuppressants with steroid monotherapy in the treatment of AIP relapses found no significant difference in relapse-free survival between the treatments ^(71)^.

### Rituximab

Retrospective studies have suggested that B-cell depletion with rituximab (an anti-CD20 antibody) is effective, even in patients for whom treatment with immunosuppressants is unsuccessful ^(71)^. In a prospective study, a disease response was observed in 97% of patients with IgG4-RD who were treated with rituximab. The effects of rituximab in IgG4-RD have been attributed, at least in part, to a failure of repletion of short-lived plasma blasts or plasma cells that produce IgG4 ^(72)^.

## Prognosis

The short-term clinical, morphological, and functional outcomes of most patients with IgG4-RD who receive steroid therapy are good ^(24)^. Steroid therapy leads to improved pancreatic endocrine and exocrine function in about half of all patients with AIP and to improved salivary and lacrimal gland function in patients with IgG4-related sialadenitis and dacryoadenitis ^(24)^.

However, long-term outcomes, including relapse, developed fibrosis, and associated malignancy, have yet to be clearly defined. For example, pancreatic juice stasis resulting from incompletely resolved narrowing of the main pancreatic duct can cause pancreatic stones ^(73)^. The risk of malignancy is also reportedly high in patients with IgG4-RD ^(74), (75)^. Although there are a few reports of AIP developing into pancreatic cancer, it is unclear whether a relationship exists between these diseases ^(24), (74)^. However, because IgG4-RD occurs predominantly in older men and steroid therapy is immunosuppressive, imaging studies, and serum tumor markers should be assessed periodically during follow-up.

## Implications

Only 18 years have passed since IgG4-RD was introduced as a novel disease entity, and the epidemiology, pathophysiology, and long-term prognosis of this disease are still unclear. Greater awareness of this protean disease is necessary to ensure an earlier diagnosis to prevent severe organ damage and disabling tissue fibrosis. The discovery of more sensitive and specific biomarkers would be a step forward in diagnosing this disease. Further, because steroid therapy involves tapering and toxicity risks, new treatment strategies, including rituximab, need to be tested in international randomized controlled clinical trials.

## Article Information

### Conflicts of Interest

None

### Sources of Funding

This work was supported by a grant from the the Japanese Ministry of Health, Labour and Welfare Research Program on Rare and Intractable Diseases (No. JPMH20FC1040).

### Acknowledgement

This work was supported by a grant from the Japanese Ministry of Health, Labour and Welfare Research Program on Rare and Intractable Diseases (No. JPMH20FC1040).

### Author Contributions

The author declares substantial contributions to the conception or design of the work, or acquisition, analysis, or interpretation of data for the work; drafting the work or revising it critically for important intellectual content; final approval of the revision to be published; and agreement to be accountable for all aspects of the work in ensuring that questions related to the accuracy or integrity of any part of the work are appropriately investigated and resolved.
